# Quality assessment of ADHD-related short videos on Chinese social media: A cross-sectional study

**DOI:** 10.1097/MD.0000000000050044

**Published:** 2026-08-07

**Authors:** Pinfang Zou, Si Chen, Junlu Xiang, Wenzhi Zhou, Xiaorong Yang

**Affiliations:** a Department of Rehabilitation, Chengdu Women’s and Children’s Central Hospital, School of Medicine, University of Electronic Science and Technology of China, Chengdu, China.

**Keywords:** attention-deficit/hyperactivity disorder (ADHD), health information, quality assessment, short videos, social media, user engagement

## Abstract

Short videos on health information related to Attention-Deficit/Hyperactivity Disorder (ADHD) on mainstream platforms in China are increasing; however, the quality of relevant content has not been systematically evaluated. A search was conducted on Douyin, Bilibili, and Xiaohongshu from August 18 to 22, 2025, using the keywords ‘Attention Deficit Hyperactivity Disorder ‘, ‘ Hyperactivity Disorder ‘, and ‘ADHD’. The top 100 videos under each keyword were collected. Two researchers independently evaluated them using GQS, mDISCERN, PEMAT-A, PEMAT-U and JAMA benchmark criteria. A total of 228 eligible videos were analyzed. Most of the videos focused on disease-related knowledge (n = 124, 54.4%) and were published by medical professionals (n = 89, 39.0%). Senior professionals had the highest content quality score (GQS median: 3). The median of mDISCERN: 2)The scores of Bilibili videos on GQS, mDISCERN and PEMAT-U were significantly higher than those of Douyin and Xiaohongshu (all *P* < .05). However, the overall information reliability (mDISCERN median: 1) and operability (PEMAT-A median: 25%) were low. It is worth noting that there was no significant positive correlation between user engagement indicators (likes, comments, sharing) and any quality score (all | *r* |<0.2), indicating weak associations with limited practical importance, while video duration was significantly but weakly positively correlated with PEMAT-A score (*R* = 0.187, *P* < .001, suggesting limited practical value). In short, although ADHD-related videos are widely prevalent on mainstream short video platforms in China, their popularity cannot fully reflect scientific validity. Overall video quality and reliability remain to be enhanced. In the future, relevant parties may consider optimizing platform algorithms, standardizing content publishers” obligations and enhancing public media literacy, to facilitate sound development of ADHD-related public health communication.

## 1. Introduction

Attention-Deficit/ Hyperactivity Disorder (ADHD) is a neurodevelopmental disorder that commonly occurs in childhood and is characterized by persistent inattention, impulsivity and hyperactivity.^[1,2]^ Even in adulthood, these symptoms may continue to be observed, which brings many problems to patients in learning, work, life and so on.^[3]^ At the same time, the patient ‘s family will also suffer great mental and economic pressure.^[4]^ Therefore, it is critical to improve the prognosis of patients by accurately identifying the early symptoms of the disease and taking correct interventions.

With the rapid development of digital new media, short-video platforms have gained tremendous popularity and become the main way for the public to obtain health information. Benefiting from massive active user groups and precise algorithmic dissemination mechanisms, an increasing number of users and medical practitioners share disease-related experience and popular science knowledge on Douyin, Bilibili, Xiaohongshu and other mainstream domestic short-video platforms.^[5,6]^ Nevertheless, a large number of unregulated self-published contents exist on these platforms, and misleading medical information will bring hidden risks to public health.^[7,8]^ In particular, inaccurate online content has been proven to greatly affect ADHD patients’ medical choices, treatment compliance and social cognition.^[9]^

Health content posted by non-professional creators tends to be less credible, a conclusion also confirmed by research on mainstream social platforms around the world: data show that about 30% to 40% of health-related videos on YouTube and TikTok contain false or misleading information.^[10–13]^ It is noteworthy that extant research on the online information of ADHD has focused primarily on international platforms (e.g., TikTok).^[14,15]^ In China, similar problems have also been observed in the research on the investigation of information quality of physical diseases (gastric cancer, inflammatory bowel disease, etc) on short-video platforms.^[16–19]^ Chinese users mainly rely on domestic social media to acquire health knowledge, and the content production, dissemination and acceptance modes present distinct local characteristics under China’s unique network environment, cultural atmosphere and information supervision system. Accordingly, research results obtained from foreign platforms cannot be directly applied to domestic situations. Most existing studies evaluate ADHD-related content using single or limited assessment tools, each of which only reflects a single dimension of content quality.^[13–15]^ Comprehensive evaluation of ADHD information therefore requires the combined use of multiple instruments. At present, there is still a lack of systematic empirical research evaluating the information quality of ADHD-related content on mainstream domestic short-video platforms.

In summary, the quality of ADHD-related short-videos on mainstream Chinese short video platforms needs to be systematically evaluated. The primary objectives of this study is to conduct a cross-sectional quality analysis of ADHD short videos across 3 major platforms: Douyin, Bilibili, and Xiaohongshu and to analyze the correlation of engagement metrics and video quality. A set of well-established assessment tools was employed to comprehensively evaluate the content characteristics and scientific rigor of the videos. These included the Global Quality Scale (GQS), the Journal of the American Medical Association (JAMA) benchmark criteria, the modified DISCERN instrument (mDISCERN), and the Patient Education Materials Assessment Tool for Actionability (PEMAT-A) and Understandability (PEMAT-U). The videos will be assessed across multiple dimensions, such as overall quality, source credibility, reliability, understandability, and actionability. The findings of this study not only provide evidence-based reference for clinicians and families of ADHD patients, but also have reference value for public health advocates and platform regulators to optimize information governance and help promote the construction of high-quality ADHD health information ecology.

## 2. Methods

### 2.1. Ethics approval

This study analyzed only publicly available short video content posted on open social media platforms (Douyin, Bilibili, and Xiaohongshu). No personal identifying information, user private data, or protected health information was accessed, collected, or processed in the course of this research. All content included in the analysis is freely accessible to the general public, and no human subjects were enrolled or interacted with. Given the nature of the data source and the absence of personal privacy concerns, ethical approval for this study was not required in accordance with relevant ethical review guidelines.

### 2.2. Data source and search strategy

The search was conducted on Douyin, Bilibili, and Xiaohongshu from August 18, 2025 to August 22, 2025. To minimize bias from personalized algorithms, we created brand-new accounts, cleared all browsing history and cache, and performed all searches on the same device under fixed network and geographic conditions to standardize settings and reduce IP‑related variation. Three Chinese keywords were used: “注意缺陷多动障碍, ” “多动症, ” and “ADHD.” Previous studies have shown that most users only browse the top-ranked search results.^[20]^ Therefore, we selected the top 100 videos per keyword per platform to reflect typical user exposure and represent the content most frequently accessed by the public.^[21]^ This approach may introduce sampling bias because the sample is limited to high-ranking videos rather than a fully random selection. Although we used new accounts and standardized search conditions, residual algorithmic bias may still exist due to inherent platform recommendation mechanisms.

### 2.3. Video screening and data extraction

Videos were included only if they met all of the following criteria:

1. Content contained information related to ADHD;
2. Its language was Chinese;
3. Its duration did not exceed 5 minutes (consistent with the standard definition of a short video);
4. The video was publicly available and open access;
5. The video could be viewed in its entirety during the data collection period.

Exclusion criteria were as follows:

1. Non-Chinese videos;
2. Duplicate videos (identified by unique video ID or identical content);
3. Purely commercial ads;
4. Videos lacking an audio track;
5. Videos exceeding 5 minutes in duration;
6. Videos with content irrelevant to ADHD.

For each eligible video, the following metadata were extracted: video URL, unique video ID, title, uploader’s username, duration (measured in seconds), upload date, and engagement metrics, including the number of likes, collections, shares, comments, and the uploader’s follower count.

The video publisher types were divided into 2 main groups: medical sources group (including junior-, intermediate-, and senior-level medical professionals) and nonmedical sources group (including parents, patients themselves, individual science communicators, and nonprofit organizations, etc).

The video content is independently coded by 2 trained researchers into the following 5 categories.: disease-related knowledge, clinical/outpatient scenarios, parenting experience, illness experience, and news reports.

### 2.4. Video quality assessment

This study used 4 validated assessment tools to comprehensively evaluate the content and quality of the videos. The GQS is a 5-point scale and was used to evaluate the overall quality and informational fluency of videos.^[21,22]^ The JAMA benchmark criteria are a widely used, reliable tool for the evaluation of health information sources and include 4 dimensions: authorship, attribution, disclosure and currency.^[23,24]^ The mDISCERN is a well-established tool for evaluating the reliability and quality of online information and consists of 5 items.^[24,25]^ Additionally, the Patient Education Materials Assessment Tool (PEMAT) was used to evaluate the actionability (PEMAT-A) and understandability (PEMAT-U) of the audiovisual materials.^[26]^ The detailed scoring criteria for each assessment tool are available in Supplementary Tables S1–S4, Supplemental Digital Content 1.

All videos were rated independently by 2 physicians (S C and Jl X) with specialized clinical expertise in ADHD. Prior to formal scoring, the raters systematically studied the scoring criteria for each tool (GQS, JAMA, mDISCERN, PEMAT-A and PEMAT-U) and unified their rating standards through independent pilot scoring and joint discussions.

All video materials were randomly ordered and distributed to the raters for independent evaluation. Inter-rater reliability was assessed using the intraclass correlation coefficient (ICC), which was calculated based on a two-way random-effects model for absolute agreement. The reliability levels were defined as follows: poor (ICC<0.50), moderate (ICC = 0.50–0.75), good (ICC = 0.75–0.90) and excellent (ICC ≥ 0.90).^[27]^ For items with initial rating discrepancies, both raters reviewed and discussed them. If they could not reach an agreement, a third senior clinical researcher (Wz Z) arbitrated to determine the final score.

The agreement between the 2 raters was good for all assessment tools. JAMA benchmark score: ICC = 0.831 (95% CI: 0.787–0.868); GQS score: ICC = 0.902 (95% CI: 0.874–0.924); mDISCERN score: ICC = 0.871 (95% CI: 0.836–0.899); PEMAT-A score: ICC = 0.905 (95%CI: 0.879–0.926); and PEMAT-U score: ICC = 0.935 (95%CI: 0.913–0.952). All ICC values were statistically significant (*P* <. 001).

### 2.5. Statistical analysis

Data reduction was performed using Microsoft 365 Excel (Microsoft, Redmond). Data analysis was performed using SPSS (Version 23.0, IBM Corp) and OriginPro (Version 2024, OriginLab Corporation). As the data were not normally distributed, descriptive statistics were presented as medians (interquartile ranges) and categorical variables were presented as frequencies (percentages). Group comparisons were conducted using the Mann–Whitney *U* test for 2 groups or the Kruskal–Wallis test for 3 or more groups. Post hoc pairwise comparisons were performed using Dunn’s test, adjusted by the Bonferroni correction. Spearman’s rank correlation analysis was used to assess correlations between variables. A two-sided *P* value of<.05 was considered statistically significant.

## 3. Results

### 3.1. The general characteristics and engagement metrics

**Figure 1.**
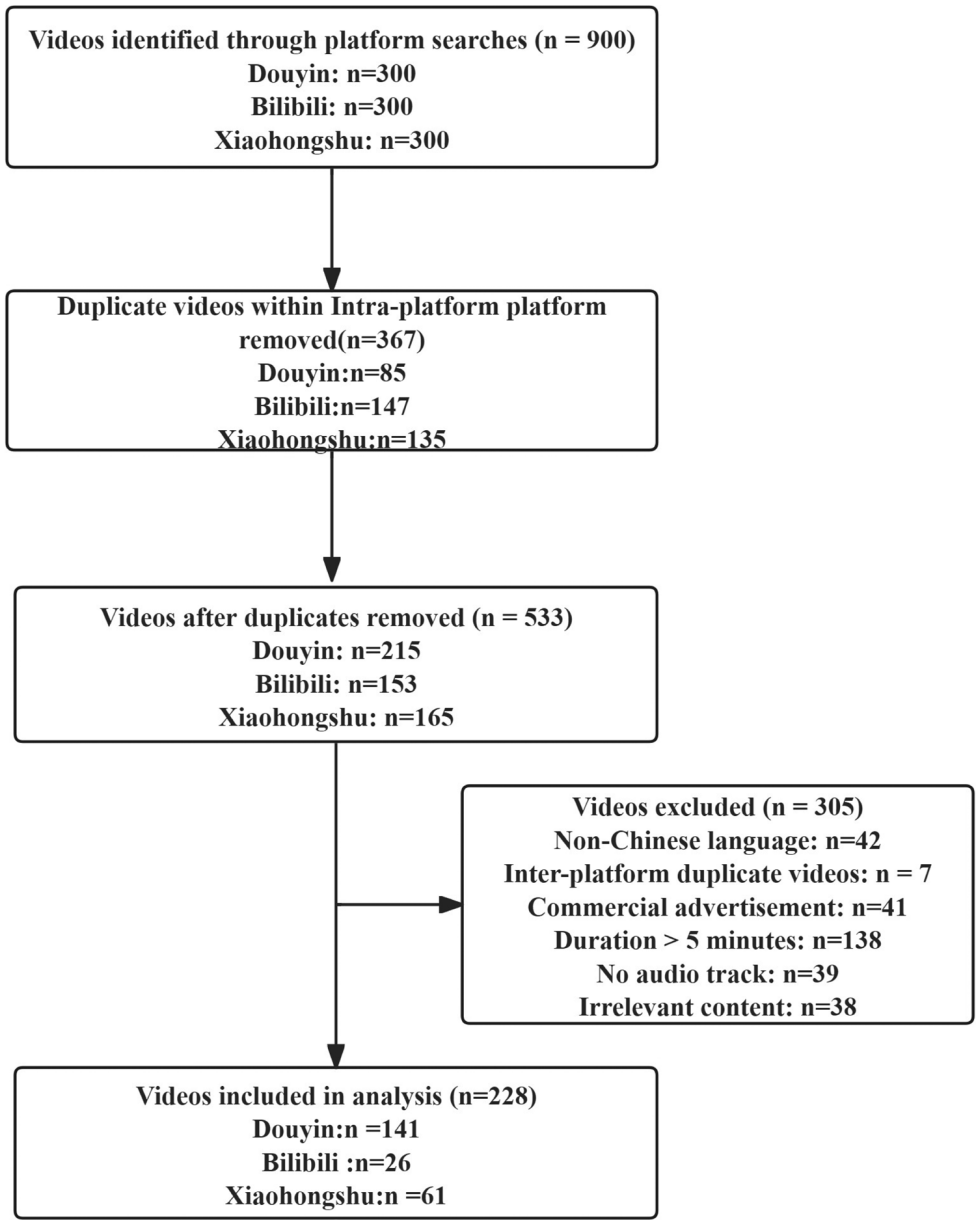
Flowchart of the study selection process for ADHD-related short videos from 3 Chinese social media platforms. The flowchart illustrates the number of videos identified, screened, and included in the final analysis. After removing duplicates and ineligible content, a total of 228 videos were included.

As shown in Figure 1, after applying the exclusion criteria, a total of 228 ADHD videos were included for analysis, with 141 videos (61.8%) sourced from Douyin, 26 videos (11.4%) from Bilibili, and 61 videos (26.8%) from Xiaohongshu. As presented in Table 1, videos from Douyin and Bilibili exhibited significantly higher interaction metrics: including likes, shares, comments, collections: and higher creator follower counts compared to videos from Xiaohongshu (all *P*<.05). No statistical difference in video duration was observed among the 3 platforms (*P*>.05).

**Table 1:** General characteristics and engagement metrics of ADHD-related videos across 3 social media platforms.

Parameters	Total (N = 228)	Douyin (n = 141)	Bilibili (n = 26)	Xiaohongshu (n = 61)	*P* value
Likes, median (IQR)	304.5 (33.0–1564.8)	446 (105–1646)	420.5 (17.8–2108.5)	29 (3–589)	< .05
Shares, median (IQR)	88.5 (9.0–576.0)	140 (22–801.5)	101.5 (7–644.5)	30 (0.5–216)	< .05
Comments, median (IQR)	49.0 (9.0–255.3)	75 (18–270)	34.5 (2–529.5)	7 (0.5–108)	< .05
Collections, median (IQR)	144.0 (21.0–629.0)	175 (43.5–737)	291 (18.8–1269.3)	23 (2–234.5)	< .05
Duration (S), median (IQR)	102.6 (64.3–169.0)	99.7 (61.4–167.5)	139.5 (87–194.5)	110 (62–165)	> .05
Fans, median (IQR)	7779.5 (1226.3–46,353.0)	10,733 (1783–46,353)	7149 (3631–95,750)	2000 (359–16,000)	< .05

*P* values were derived from the Kruskal–Wallis test. Statistical significance was set at *P* < .05. Across platforms, Douyin videos showed the highest median values for likes, shares, comments, and creator follower counts, while Xiaohongshu videos had the lowest engagement. Bilibili videos had the highest median collection counts. Video duration did not differ significantly among the 3 platforms.

IQR = interquartile range, S = Seconds.

### 3.2. Distribution of video publisher types and content across platforms

**Figure 2.**
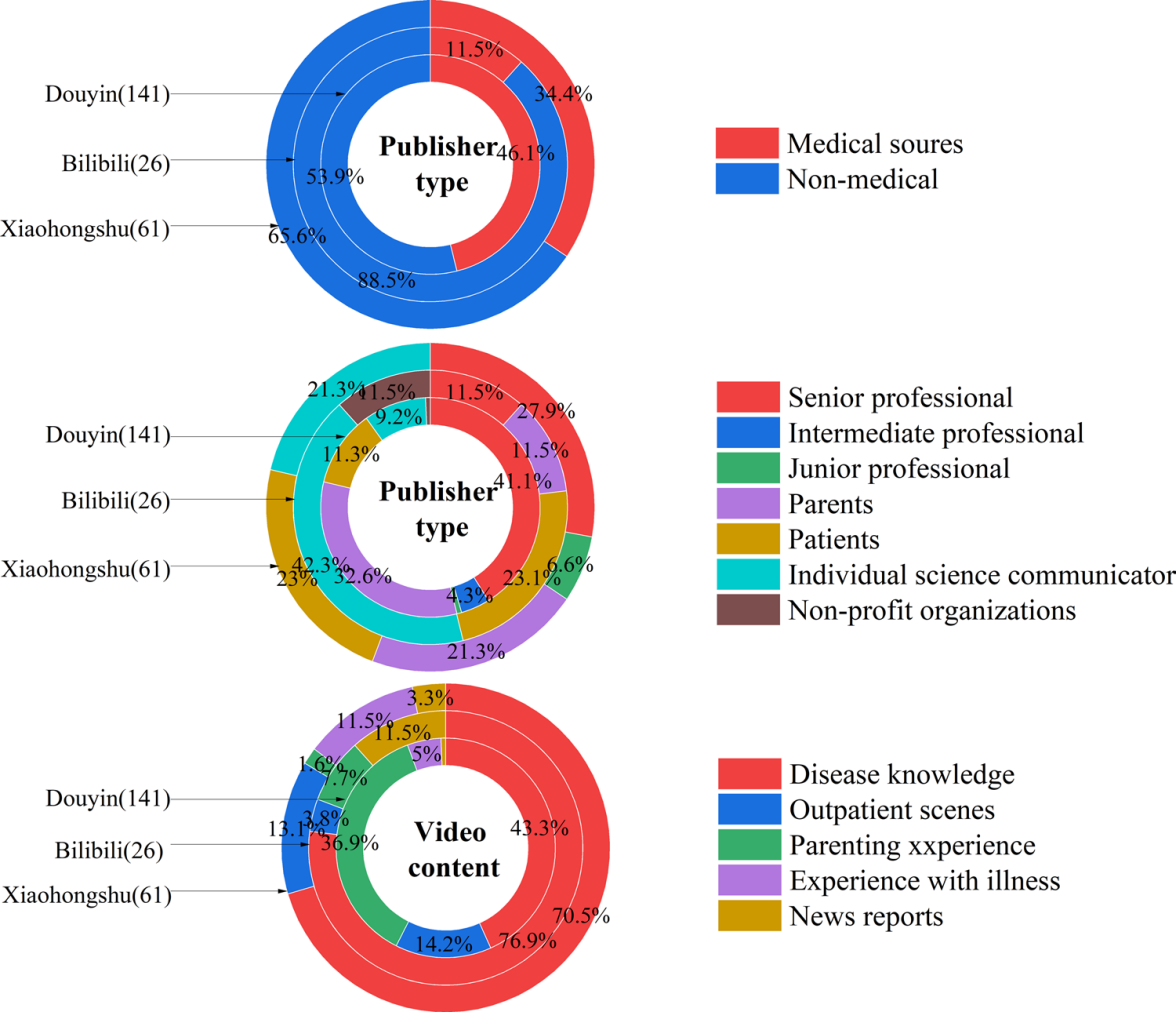
Distribution of ADHD video themes across 3 platforms. Parenting experience videos were most common on Douyin, while knowledge-based content dominated on Bilibili.

As shown in Figure 2, medical professionals were the main publishers of the videos (89/228; 39.0%), most of whom held senior professional positions (78/89; 87.6%). The proportion of videos published by senior medical professionals was the highest on Douyin (58/141, 41.1%). Among nonmedical sources, the largest subgroup was parents of patients (62/139, 44.6%), followed by individual science communicators (37/139, 26.6%) and patients themselves (36/139, 25.9%). The proportion of individual science communicators was the highest on Bilibili (11/26, 42.3%).

In terms of video content, the most prevalent topic was disease knowledge (124/228, 54.4%), followed by parenting experience (55/228, 24.1%) and clinical/outpatient scenarios (29/228, 12.7%). Disease knowledge was the dominant theme on both Bilibili (20/26, 76.9%) and Xiaohongshu (43/61, 70.5%), whereas a substantial proportion of videos on Douyin focused on parenting experience (52/141, 36.9%).

### 3.3. Video quality across platforms

**Figure 3.**
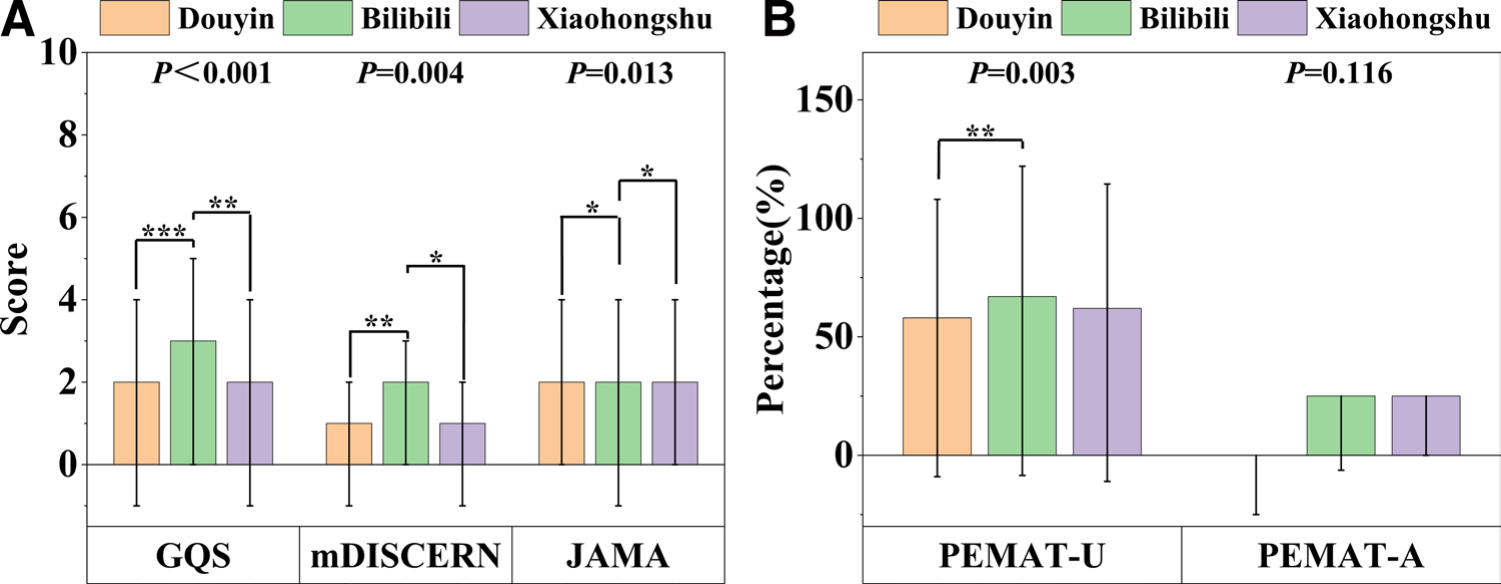
(A) Comparison of quality scores of ADHD-related videos across 3 platforms (Douyin, Bilibili, Xiaohongshu). Data are presented as median (IQR). Positive and negative error bars indicate the IQR bounds, respectively. Post hoc pairwise comparisons were performed using the Dunn test, with P-values adjusted via the Bonferroni method. *, ** and *** indicate significance at the 0.05, 0.01 and 0.001 probability levels, respectively. (B) Bilibili videos showed significantly higher quality scores across most indicators compared with Douyin and Xiaohongshu, while no significant between-platform difference was observed in actionability (PEMAT-A). GQS = Global Quality Scale, JAMA = Journal of the American Medical Association (benchmarks), mDISCERN = modified DISCERN instrument, PEMAT-A = Patient Education Materials Assessment Tool for Actionability, PEMAT-U = Patient Education Materials Assessment Tool for Understandability.

The quality of ADHD videos varied significantly across the 3 social media platforms (Fig. 3). The Kruskal–Wallis test revealed significant differences in the GQS, mDISCERN, PEMAT-U, and JAMA scores between Douyin, Bilibili, and Xiaohongshu (all *P* < .05), but no significant difference was observed in the PEMAT-A score (*P*=.116). Post hoc Dunn tests showed that videos on Bilibili had significantly higher GQS scores than those on Douyin (adjusted *P* < .001) and Xiaohongshu (adjusted *P* = .010). Similarly, videos on Bilibili had significantly higher mDISCERN, PEMAT-U, and JAMA scores than those on the other 2 platforms in pairwise comparisons (all adjusted *P* < .05).

### 3.4. Video quality by publisher type and content

**Figure 4.**
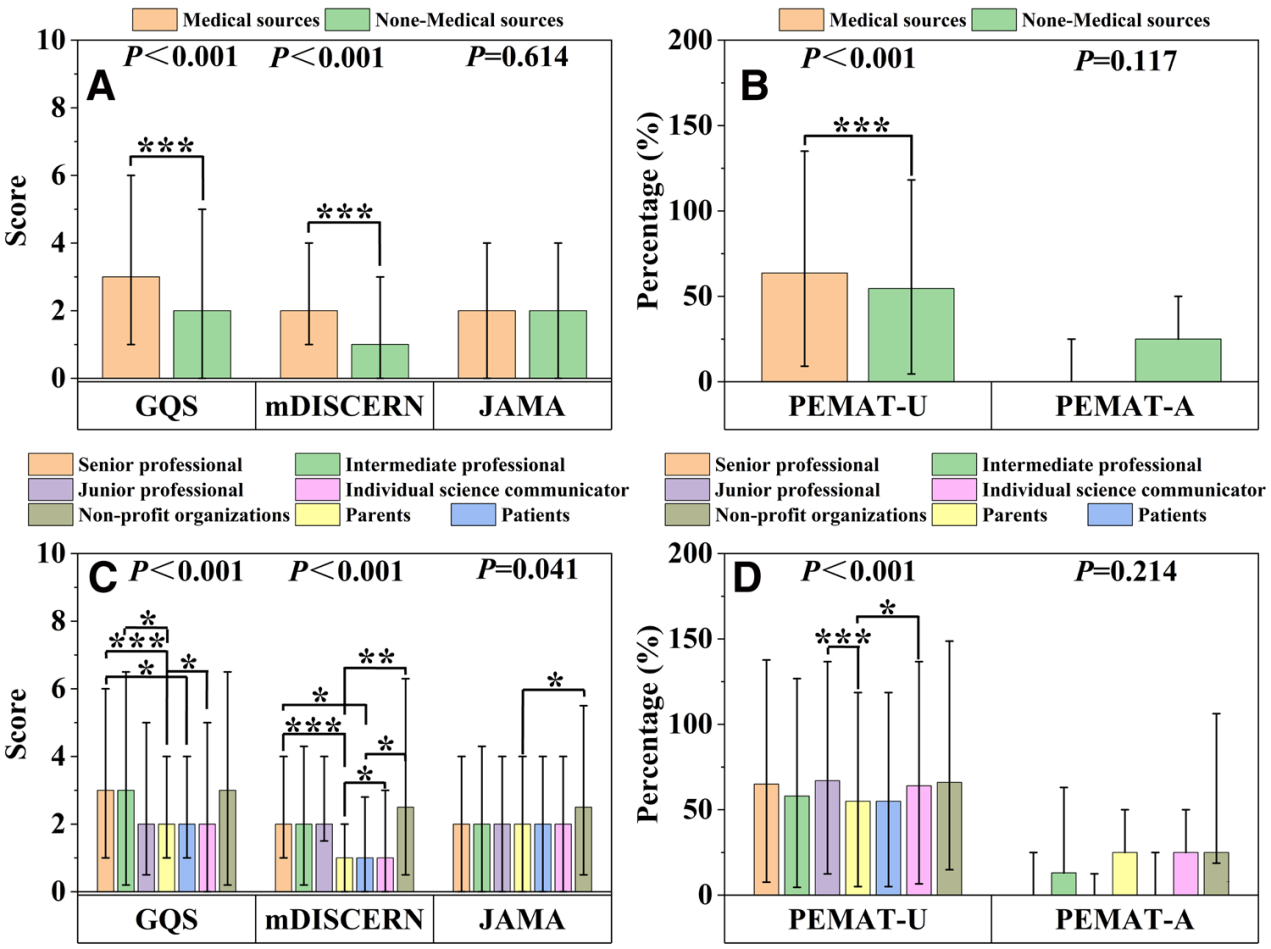
Video quality assessed by GQS, mDISCERN, JAMA (A, C) and PEMAT-U, PEMAT-A (B, D) across different publisher types. Data are presented as median (IQR). Positive and negative error bars indicate the IQR bounds, respectively. Post hoc pairwise comparisons were performed using the Dunn test, with P-values adjusted via the Bonferroni method. *, ** and *** indicate significance at the 0.05, 0.01 and 0.001 probability levels, respectively. Nonmedical contributors accounted for the largest share of publishers overall, while Douyin had the highest proportion of videos from medical professionals. GQS = Global Quality Scale, JAMA = Journal of the American Medical Association (benchmarks), mDISCERN = modified DISCERN instrument, PEMAT-A = Patient Education Materials Assessment Tool for Actionability, PEMAT-U = Patient Education Materials Assessment Tool for Understandability.

**Figure 5.**
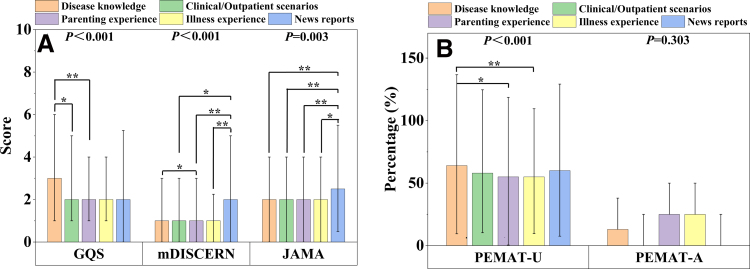
Video quality assessed by GQS, mDISCERN, JAMA (A) and PEMAT-U, PEMAT-A (B) across different video content. Data are presented as median (IQR). Positive and negative error bars indicate the IQR bounds, respectively. Post hoc pairwise comparisons were performed using the Dunn test, with *P*-values adjusted via the Bonferroni method. *, ** and *** indicate significance at the .05, .01 and .001 probability levels, respectively. Videos from medical professionals showed significantly higher quality scores than those from nonmedical contributors.GQS = Global Quality Scale, JAMA = Journal of the American Medical Association (benchmarks), mDISCERN = modified DISCERN instrument, PEMAT-A = Patient Education Materials Assessment Tool for Actionability, PEMAT-U = Patient Education Materials Assessment Tool for Understandability.

A substantial variation in video quality was identified among diverse publisher categories (Fig. 4) and content themes (Fig. 5) (all *P* < .05).

Publisher type: In general, videos from medical sources achieved significantly higher scores in the GQS, mDISCERN, and PEMAT-U assessments than those from nonmedical sources (*P* < .001). Among medical sources, videos produced by professionals in senior positions received the highest median scores in terms of overall quality (GQS score: 3), reliability (mDISCERN score: 2), and understandability (PEMAT-U score: 64%), thus demonstrating superiority over other subgroups (all *P* < .001). Nonprofit organizations also obtained notably high scores on several metrics (e.g., mDISCERN score: 2.5). Conversely, videos from nonmedical publishers (e.g., parents, patients, individual communicators) exhibited lower performance across these core quality indicators.

Video content: Disease knowledge received the highest median scores in core quality metrics (GQS score: 3; PEMAT-U score: 64%). News report achieved the highest scores in the JAMA benchmark (median score: 2.5) while having lower reliability scores (mDISCERN score: 2). Significant variations were identified in the GQS, mDISCERN, PEMAT-U, and JAMA scores across different content themes (*P* < .01). Nevertheless, no significant differences were observed in the action ability domain (PEMAT-A score; *P*>.05).

### 3.5. Correlation between engagement metrics and video quality

**Figure 6.**
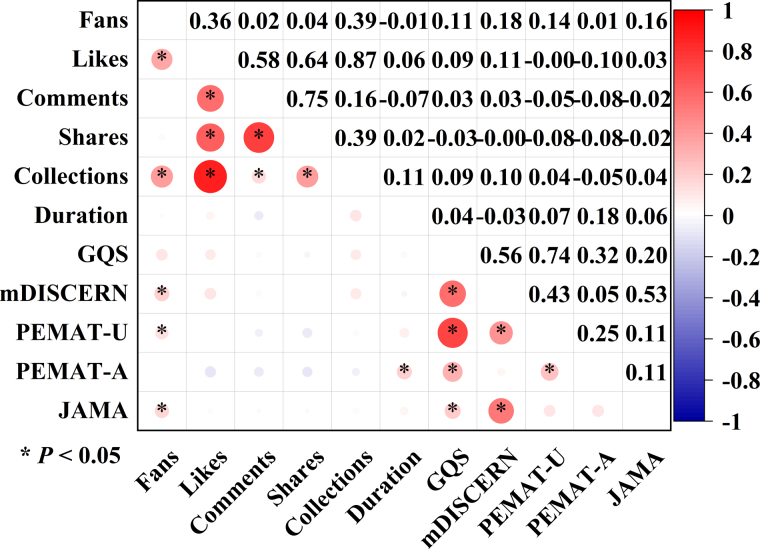
Heatmap of Spearman correlations between engagement metrics and quality scores of ADHD-related videos. Color intensity denotes correlation strength/direction (*r*); red = positive, blue = negative. Upper-triangle values are correlation coefficients (*r*). * indicates significance at *P* < .05. Engagement metrics (likes, comments, shares, collections) showed weak or no correlation with quality scores (|*r*|<0.2), indicating that video popularity was not associated with scientific quality. GQS = Global Quality Scale, JAMA = Journal of the American Medical Association (benchmarks), mDISCERN = modified DISCERN instrument, PEMAT-A = Patient Education Materials Assessment Tool for Actionability, PEMAT-U = Patient Education Materials Assessment Tool for Understandability.

As demonstrated in Figure 6, Spearman correlation analysis revealed significant relationships among the video variables. The analysis revealed moderate to strong positive correlations between engagement metrics. The data demonstrated a strong positive correlation between “likes” and ‘collections’ (*R* = 0.87, *P* < .001) and a moderate positive correlation between “comments” and ‘shares’ (*R* = 0.75, *P* < .001). Within the quality assessment tools, the GQS score demonstrated a strong positive correlation with understandability (PEMAT-U) (*R* =0.74, *P* < .001), and the modified DISCERN (mDISCERN) score exhibited a moderate correlation with the abbreviated JAMA score (*R* = 0.53, *P* < .001). However, no significant correlations were identified between engagement metrics (i.e., likes, comments, shares, collections, follower count) and quality scores (GQS, mDISCERN, PEMAT-U, PEMAT-A, JAMA) (|*r*|<0.2), indicating weak or negligible associations with limited practical significance. It is noteworthy that the number of comments exhibited a weak negative correlation with understandability (*r* = −0.20, *P*=.002), while longer video duration showed a statistically significant but weak positive correlation with higher actionability (*R* = 0.19, *P*<.001), with limited clinical or practical relevance.

## 4. Discussion

### 4.1. Principal findings

ADHD is a common neurodevelopmental condition that is becoming an increasingly prevalent public health concern. Short video platforms have emerged as a key channel for the public to access health information.^[28,29]^ Previous studies have demonstrated substantial variability in the quality of health information across different platforms and sources.^[17,30]^ However, there has been a lack of systematic evaluation of the quality of ADHD videos on mainstream Chinese social media platforms. To address this, this study conducted a cross-sectional analysis of ADHD content on 3 major platforms: Douyin, Bilibili and Xiaohongshu.

The study found that videos on Bilibili had significantly higher scores in GQS, mDISCERN, and PEMAT-U than those on Douyin and Xiaohongshu. However, the overall PEMAT-A score for all platforms is generally low (median: 25%), and the overall reliability of the information provided is suboptimal (mDISCERN median: 1). The medical group, especially senior-level medical professionals, contributed most of the high-quality content, and their video quality was significantly better than that created by nonprofessionals (all *P*<.001). In terms of video content, the “disease knowledge” video had the highest quality, while the “parenting experience” video was the most popular on Douyin, accounting for 36.88%. A key finding of this study was that there was no significant positive correlation between engagement indicators (such as likes, comments, sharing, collections, etc) and science quality scores, which differs from the observation results of some other disease studies (|*r|*<0.2). These results indicated that the quality reliability of ADHD-related information was not closely related to its transmission popularity.

### 4.2. Video popularity and dissemination

Previous studies have shown that the number of likes, comments, collections, and shares are core indicators that reflect the popularity of video.^[30,31]^ This study found that some ADHD-related videos have received hundreds of thousands of likes and tens of thousands of collections and shares, which reflects a lot of public attention to ADHD. With its large and diverse user base and mature algorithm recommendation system, Douyin shows a significant lead in likes, comments, sharing, etc, which highlights its strong capacity to amplify content reach. In contrast, Bilibili has an advantage in the number of collections, reflecting the users’ deep exploration of the value of content, which is mainly related to the strong knowledge community atmosphere created by the Bilibili platform. Compared with the other 2 platforms, Xiaohongshu performs worse on all participation indicators, suggesting its community attribute is not conducive to the dissemination of this research content.

The interaction between publisher type and platform attributes is worthy of attention. Although nonmedical professionals account for the highest proportion of video publishing, the highest proportion of video publishing on the Douyin platform is medical professionals (with the highest proportion of senior titles). These results show that there are cognitive differences in the choice of video publishing platform among different publisher types.^[32]^ In contrast, the high proportion of “individual science communicators” on Bilibili (42.31%) aligns with the platform’s community culture, in which users actively seek systematic, knowledge-based content. The driving factors behind the spread of different content themes need to be understood in combination with the specific characteristics of the disease. This study found that ‘parenting experience ‘ videos accounted for a relatively high proportion (36.88%) in Douyin. These videos have a high degree of participation, which is mainly related to their ability to meet the needs of ADHD caregivers (mainly parents) for medical knowledge, emotional resonance, sense of community belonging, and practical strategies to deal with daily challenges.^[2]^ Research indicates that families of children with ADHD have faced various challenges that are underestimated by society for a long time, including education system pressure, parenting pressure, children ‘s social dilemma, psychological burden, etc^[33,34]^ Therefore, the behavior of parents sharing ‘ parenting experience ‘ on short video platforms essentially creates a convenient and accessible online peer support network, providing an outlet for emotional expression and a medium for sharing experiences for originally isolated families. However, such experiential content may lack sufficient scientific evidence and could inadvertently spread non–evidence-based information, which warrants public caution.^[13,35]^

### 4.3. Video quality

This study further clarifies the factors related to video quality. Firstly, uploader identity is a key factor affecting video quality. The quality-related indicators of videos produced by senior medical professionals (*P*<.001) were significantly better than those of nonprofessionals, which was consistent with international findings that the content produced by medical service providers is usually more professional and accurate.^[14,36]^ Secondly, platform attributes are also a key factor affecting the quality of video content. Specifically, this study found that Bilibili was significantly better than Douyin and Xiaohongshu in many quality indicators, which may be due to its community ecology that encourages in-depth and systematic content creation. Therefore, for ADHD, which requires long-term management and professional guidance, Bilibili may be more conducive to the effective communication of such health knowledge. This finding is also consistent with the conclusions of short video studies on other health issues, such as gastrointestinal tumors.^[17,30]^ Noticeably, evident quality disparities also exist among videos of different themes: videos focusing on standardized disease knowledge obtain higher quality scores than those centered on personal experience sharing. Such experiential content lacks sufficient scientific rigor, and blind credence in relevant personal narratives may easily mislead patients and their families, a hidden risk also mentioned in overseas studies on ADHD online health content.^[13–15]^ In addition, research shows that platform format constraints such as video duration are important factors restricting information operability. This study confirmed a significant positive correlation between video duration and PEMAT-A scores (*R* = 0.187, *P* < .001, suggesting limited practical value), which is consistent with other health communication research.^[14,32]^ The short duration of most videos (half of the videos included in this study were under 1 minute) makes it extremely difficult to include specific, step-by-step behavioral guidance or detailed healthcare navigation advice for complex conditions such as ADHD. Therefore, low “actionability” is a common challenge of the short-video format for disseminating complex health information.

### 4.4. Correlation of engagement metrics and video quality

The correlation analysis in this study revealed a critical and cautionary finding: there was no significant positive correlation between video popularity metrics (such as likes, comments, shares, and collections) and any of the scientific quality indicators (|*r*|<0.2). This result challenges the common assumption that “high engagement equates to high informational value,” suggesting that in the context of ADHD, the logic underlying the widespread dissemination of content is distinct from that ensuring scientific rigour.

This finding is consistent with a number of recent research conclusions, that is, emotional resonance or misleading information is more likely to obtain algorithm recommendations and user interaction.^[13,35–37]^ This phenomenon may be attributed to the algorithmic orientation of mainstream platforms with user interaction as the core. The design goal of such algorithms is mainly to extend the user ‘s stay time and improve interactive indicators such as likes, comments, and sharing, rather than evaluating the scientific quality of the content. Therefore, some content with insufficient scientific basis but strong narrative and prominent emotional impact can often obtain exposure opportunities far beyond its actual scientific value.^[13,30]^ This phenomenon has a profound impact on public health. Studies have confirmed that exposure to ADHD-related misinformation on social media weakens the public’s accurate understanding of the disease and increases the risk of self-diagnosis or inappropriate medical behavior. The potential link to the Dunning-Kruger effect remains interpretive and lacks direct empirical support in this study, so it is not further emphasized.^[38]^ In addition, long-term exposure to such content may also distort the public’s perception of the true prevalence of the disease, forming an information ‘ echo chamber ‘, thereby exacerbating the solidification and dissemination of misconceptions.^[13,39]^ It is worth noting that the Bilibili platform showed significantly better video quality in this evaluation. This means that algorithm design may take into account the long-term value of user immediate feedback and content. The above findings have some implications for optimizing the online environment of health information, but in view of the small sample size included in the platform, the conclusion still needs to be verified by larger-scale studies.

### 4.5. Recommendations

These findings have clear and actionable public health implications. First, healthcare professionals should play a more active role in producing and disseminating evidence-based ADHD content to improve the overall quality of online information. Second, structured digital health literacy programs are needed to help the public critically evaluate online health information and avoid overreliance on engagement metrics. Third, platforms may consider implementing appropriate content verification strategies for health-related topics, such as creator credential verification and clear labeling of experiential content, and further optimize medical content recommendation mechanisms to increase exposure of high-quality professional popular science content.

Given the high professional threshold of ADHD-related popular science knowledge, such professional content is hard to fit the entertaining expression style of mainstream short videos, restricting its wide public dissemination. In this context, both professional creators and ordinary experience sharers are suggested to enrich videos with specific and operable practical guidance. Experience-sharing creators should clearly distinguish personal perceptions from formal medical facts, and add explicit reminders to consult professional clinicians. For the general public, it is essential to remain cautious toward emotional parenting and disease experience videos, verify creators’ professional qualifications rationally, and treat relevant online content merely as auxiliary reference rather than the basis for clinical medical decisions.

### 4.6. Limitations

There are some limitations in this study. First of all, the video data came from China’s Douyin, Bilibili and Xiaohongshu, and social media platforms with other language or cultural backgrounds might not be able to directly use the results of this study. Second, the number of video samples of the 3 platforms was unevenly distributed, and the sample size of Bilibili was relatively small, which might affect the stability and representativeness of some statistical results. Thirdly, although several widely used assessment tools were selected in this study, and the consistency assessment of the independent assessment scores of the 2 raters was conducted, it was undeniable that these assessments were still subjective. Fourth, this study adopted a cross-sectional design that only captured data at a single time point, which can only reflect associations rather than causal relationships; thus, causal inference between video exposure and audience cognition or behavior is limited. Finally, in order to reduce the interference of personalized recommendations, although this study used new accounts, the inherent recommendation mechanism of the platform was still difficult to eliminate. Specifically, platform algorithms may still prioritize trending or high-engagement content rather than scientifically rigorous information; the search-based sample in this study may not fully represent the personalized feeds that individual users normally view; and regional or temporal variations in content ecology and algorithm rules may also reduce the representativeness of the sampling, which might affect the representativeness of the final sample.

## 5. Conclusion

Although ADHD-related short videos are widely popular on mainstream Chinese social media platforms, their scientific value cannot be accurately judged by user engagement alone, and overall quality and reliability require improvement. Findings suggest the need for improved content quality and reliability of ADHD-related health information on these platforms. Future research and platform collaboration may help address these gaps by optimizing recommendation algorithms, clarifying content creator responsibilities, and enhancing public digital health literacy, thereby supporting sound development of ADHD public health communication. This cross‑platform analysis demonstrates that videos were primarily posted by medical professionals (especially senior-level providers), with disease knowledge as the dominant theme. Nevertheless, overall quality varied widely, and actionability scores were consistently low. While experiential content such as parenting experience was widely disseminated, it may carry potential risks of inaccuracy. Our findings further confirm that high video popularity does not equate to stronger scientific reliability. The public should remain cautious when viewing ADHD-related online content and prioritize information from verified professional sources. Platforms and creators are encouraged to work together to improve the accuracy and practical value of health information.

## Acknowledgments

The authors are grateful for the relevant contributions of all individuals.

## Author contributions

**Project administration:** Pinfang Zou, Xiaorong Yang.

**Supervision:** Pinfang Zou, xiaorong Yang.

**Formal analysis:** Si Chen, Xiaorong Yang.

**Investigation:** Si Chen.

**Data curation:** Junlu Xiang.

**Methodology:** Wenzhi Zhou.

**Conceptualization:** Xiaorong. Yang.

**Writing – review & editing:** Pinfang Zou, Xiaorong Yang.








