# Supplementary material for: Quality assessment of ADHD-related short videos on Chinese social media: A cross-sectional study
Source: Medicine (Baltimore). 2026 Aug 7;105(32):e50044. doi: 10.1097/MD.0000000000050044 (PMC13456716; doi:10.1097/MD.0000000000050044)
Supplement: Supplementary file 1 [file medi-105-e50044-s001.docx]

**Supplementary**

**Table S1. Description of the Global Quality Scale (GQS).**

| Scale | Description |
| --- | --- |
| Poor quality (1 point) | Poor quality and poor flow of the site, most information missing, not at all useful for patients |
| Generally poor quality (2 point) | Generally poor quality and poor flow, some information listed but many important topics missing, of very limited use to patients |
| Moderate quality (3 point) | Moderate quality, sub-optimal flow, some important information is adequately discussed but others poorly discussed, somewhat useful for patients |
| Good quality (4 point) | Good quality and generally good flow, most of the relevant information is listed, but some topics not covered, useful for patients |
| Excellent quality (5 point) | Excellent quality and excellent flow, very useful for patients |
