# Supplementary material for: Quality assessment of ADHD-related short videos on Chinese social media: A cross-sectional study
Source: Medicine (Baltimore). 2026 Aug 7;105(32):e50044. doi: 10.1097/MD.0000000000050044 (PMC13456716; doi:10.1097/MD.0000000000050044)
Supplement: Supplementary file 2 [file medi-105-e50044-s002.docx]

**Supplementary**

**Table S2. The Journal of American Medical Association (JAMA) benchmark criteria.**

| Criteria  (1 point for each) | Description |
| --- | --- |
| Authorship | Author and contributor credentials and their affiliations should be provided |
| Attribution | Clearly lists all copyright information and states references and sources for content |
| Currency | Initial date of posted content and subsequent updates to content should be provided |
| Disclosure | Conflicts of interest, funding, sponsorship, advertising, support, and video ownership should be fully disclosed |
