# Supplementary material for: Quality assessment of ADHD-related short videos on Chinese social media: A cross-sectional study
Source: Medicine (Baltimore). 2026 Aug 7;105(32):e50044. doi: 10.1097/MD.0000000000050044 (PMC13456716; doi:10.1097/MD.0000000000050044)
Supplement: Supplementary file 3 [file medi-105-e50044-s003.docx]

**Supplementary**

**Table S3. Modified DISCERN (mDISCERN) scoring standard. (1 point for answer ‘yes’, 0 point for answer ‘no’)**

| Description | Reliability Score |
| --- | --- |
| 1. Is the video clear, concise, and understandable? |  |
| 2. Are valid sources cited? |  |
| 3. Is the content presented balanced and unbiased? |  |
| 4. Are additional sources of content listed for patient reference? |  |
| 5. Are areas of uncertainty mentioned? |  |
