# Supplementary material for: Quality assessment of ADHD-related short videos on Chinese social media: A cross-sectional study
Source: Medicine (Baltimore). 2026 Aug 7;105(32):e50044. doi: 10.1097/MD.0000000000050044 (PMC13456716; doi:10.1097/MD.0000000000050044)
Supplement: Supplementary file 4 [file medi-105-e50044-s004.docx]

**Supplementary**

**Table S4. The Patient Education Materials Assessment Tool for Understandability (PEMAT-U) and Actionability (PEMAT-A)**

**Understandability.**

| Item # | Item | Response Options | Rating |
| --- | --- | --- | --- |
| Topic: Content | | | |
| 1 | The material makes its purpose completely evident. | Disagree=0, Agree=1 |  |
| Topic: Word Choice & Style | | | |
| 3 | The material uses common, everyday language. | Disagree=0, Agree=1 |  |
| 4 | Medical terms are used only to familiarize audience with the terms. When used, medical terms are defined. | Disagree=0, Agree=1 |  |
| 5 | The material uses the active voice. | Disagree=0, Agree=1 |  |
| Topic: Organization | | | |
| 8 | The material breaks or "chunks" information into short sections. | Disagree=0, Agree=1,  Very  short material=N/A |  |
| 9 | The material’s sections have informative headers. | Disagree=0, Agree=1,  Very  short material=N/A |  |
| 10 | The material presents information in a logical sequence. | Disagree=0, Agree=1 |  |
| 11 | The material provides a summary. | Disagree=0, Agree=1,  Very  short material=N/A |  |
| Topic: Layout & Design | | | |
| 12 | The material uses visual cues (e.g., arrows, boxes, bullets, bold, larger font, highlighting) to draw attention to key points. | Disagree=0, Agree=1, Video=N/A |  |
| 13 | Text on the screen is easy to read. | Disagree=0, Agree=1,  No text or all text is narrated=N/A |  |
| 14 | The material allows the user to hear the words clearly (e.g., not too fast, not garbled). | Disagree=0, Agree=1,  No narration=N/A |  |
| **Topic: Use of Visual Aids** | | | |
| 18 | The material uses illustrations and photographs that are clear and uncluttered. | Disagree=0, Agree=1,  No visual aids=N/A |  |
| 19 | The material uses simple tables with short and clear row and column headings. | Disagree=0, Agree=1,  No tables=N/A |  |

Total Points: _____________

Total Possible Points: _____________

Understandability Score (%): _____________

(Total Points / Total Possible Points x 100)

**Actionability**

| **Item#** | **Item** | **Response Options** | **Rating** |
| --- | --- | --- | --- |
| 20 | The material clearly identifies at least one action the user can take. | Disagree=0, Agree=1 |  |
| 21 | The material addresses the user directly when describing actions. | Disagree=0, Agree=1 |  |
| 22 | The material breaks down any action into manageable, explicit steps. | Disagree=0, Agree=1 |  |
| 25 | The material explains how to use the charts, graphs, tables, or diagrams to take actions. | Disagree=0, Agree=1,  No charts, graphs, tables, diagrams=N/A |  |

Total Points: _____________

Total Possible Points: _____________

Actionability Score (%): _____________

(Total Points / Total Possible Points x 100)
